# Abstracts

**DOI:** 10.1155/S1463924682000273

**Published:** 1982


### Abstracts

### Page 112

### Automated atomic absorption determination of lead in gasoline

J. H. Lowry *et al.*

A comparison between a newly developed automated method and a legally acceptable method for the analysis of lead in gasoline is presented. The effect of storage conditions and holding time by container type in the lead content of gasoline has also been investigated.

### Dosage automatisé du plomb dans l'essence par absorption atomique

J. H. Lowry *et al.*

Une comparaison entre cette nouvelle méthode automatique et la méthode légale reconnue pour l'analyse du plomb dans l'essence est présentée.

Les effets des conditions de conservation des échantillons d'essence contenant du plomb sont aussi étudiés.

### Automatisierte Bestimmung von Blei in Benzin mit Atom-Absorption

J. H. Lowry *et al.*

Eine neu entwickelte, automatisierte Methode für die Bestimmung von Blei in Benzin wird mit einer rechtlich anerkannten Methode verglichen. Dazu wird der Einfluss der Lagerbedingungen und der Lagerdauer für verschiedene Lagerbehälter auf den Bleigehalt untersucht.

---

### Page 116

### An analyser for the continuous determination of acrolein in the atmosphere

J. F. Reddish

Concern about the toxic and irritant effects of aldehydes, particularly acrolein, produced during degradation or combustion of plastics has resulted in the development of a specific analyser for monitoring acrolein on a continuous basis during processing and during combustion or high-temperature degradation experiments. The analyser relies on continuous atmosphere sampling and reaction of the acrolein with a reagent containing 4-hexylresorcinol under optimum conditions to form a coloured complex which is measured in a flow-through cell. The effective range of the analyser is 0·01 to 17 ppm acrolein in air and a result is obtained within a delay time of 15–20 min. after air sampling has started. The reaction is virtually specific for acrolein.

### Un analyseur pour le dosage en continu de l'actoleine dans l'atmosphere

J. F. Reddish

A propos des effets toxiques et irritants des aldéhydes, et plus particulièrement de l'acroléine, produite durant la dégradation ou la combustion des plastiques, il a été développé un analyseur spécifique pour surveiller en continu la production d'acroléine durant les expériences de dégradation soit en combustion lente, soit à haute température. L'analyseur est basé à partir d'un échantillonage en continu de l'atmosphère sur la réaction de l'acroléine avec un réactif contenant de la 4-hexylresorcinol ceci dans des conditions optimums pour former un complexe coloré et qui est mesuré dans une cuve à circulation. La zone de dètection efficace d'un analyseur est comprise entre 0,01 à 17 ppm d'acroléine contenue dans l'air, et le résultat est obtenu après un délai de 15–20 minutes après le début du prélèvement d'atmosphère. La réaction est virtuellement specifique de l'acroléine.

### Analysator für die kontinuierliche Bestimmung von Akrolein in der Atmosphäre

J. F. Reddish

Die Besorgnis über die toxische und irritierende Wirkung von Aldehyden, besonders Akrolein, die während des Abbaus oder der Verbrenung von Kunststoffen entstehen, resultierten in der Entwicklung eines spezifischen Analysators für die Ueberwachung von Akrolein auf kontinuierlicher Basis während der Verarbeitung und der Verbrennung oder bei Abbau-Versuchen unter erhöhten Temperaturen. Der Analysator basiert auf einer kontinuierlichen Probenahme aus der Atmosphäre und Reaktion des Akroleins mit einem Reagens, das 4-Hexylresorcinol enthält und unter optimalen Bedingungen einen Farbkomplex bildet, der in einer Durchflusszelle vermessen wird. Der effektive Bereich des Analysators geht von 0,01–17 ppm Akrolein in Luft, und ein Resultat wird innerhalb von 15–20 Minuten erhalten, nachdem mit der Probenahme aus der Luft begonnen wurde. Die Reaktion ist buchstäblich spezifisch für Akrolein.

---

### Page 122

### An assessment of the performance of the Chemispek J 200 in a hospital laboratory

G. Dale *et al.*

The Chemispek J 200 is a recently introduced continuous-flow multichannel autoanalyser. It comprises of several interconnecting modules: sampler, gearbox and pumps, recorder, microprocessor, printer, cassette-recorder and power-pack. A six-channel instrument is used for the analysis of sodium, potassium, chloride, bicarbonate, urea and creatinine at a sample rate of 120/h using approximately 140 $\mu$l of sample. The chemistries used are flame photometry for sodium and potassium, thiocyanate-nitrate for chloride, cresol red for bicarbonate, diacetylminoxime for urea and alkaline picrate for creatinine. The microprocessor standardizes, calculates, corrects for drift and carry-over and the six-pen recorder monitors the peaks. Analyte concentrations spanned the range of 100 to 154 mmol/l of sodium, 2 to 10 mmol/l of potassium, 84 to 130 mmol/l of chloride, 10 to 40 mmol/l of bicarbonate, 3·5 to 30 mmol/l of urea and 20 to 1000 $\mu$mol/l of creatinine. Within-run imprecision CVs ranged from 0·37% to 4·9%, between imprecision CVs ranged from 0·88% to 5·92%. Comparison of the assayed results and the consensus results from two external quality assurance schemes showed excellent agreement for all six chemistries. During the evaluation period a number of mechanical and constructional problems were encountered and resolved by the manufacturer. Imprecision and comparative quality-assurance data indicate that the Chemispek J 200 measures the analytes (sodium, potassium, chloride, bicarbonate, urea, creatinine) with an excellent degree of precision and accuracy.

### Une évalutation des performances du Chemispek J 200 dans un laboratoire hospitalier

G. Dale *et al.*

Le Chemispek J 200 est un analyseur flux continu multicanaux. Il comprend plusieurs modules interconnectés: préleveur d'échantillon, pompes à vitesse variable, enregistreur, microprocesseur, imprimante, enregistreur de cassette et module d'alimentation.

Cet instrument à six canaux est utilisé pour l'analyse des paramètres suivants: sodium, potassium, chlorures, bicarbonates, urée et créatinine; la cadence est de 120 échantillons à l'heure pour un prélèvement approximatif de 140 μl par échantillon.

Les méthodologies utilisées sont: la photométrie de flamme pour le sodium et le potassium, le thiocyanate-nitrate pour les chlorures, le rouge de crezol pour les bicarbonates, la diacétylmonoxime pour l'urée et le picrate alcalin pour la créatinine.

Le microprocesseur pilote l'enregistreur 6 voies et à partir des pics étalonne et calcule les résultats, corrige les effets de détive et de contamination.

La zone de concentration étudiée est: entre 100 et 154 mmol/l pour le sodium, entre 2 et 10 mmol/l pour le potassium, entre 84 et 130 mmol/l pour les chlorures, entre 10 et 40 mmol/l pour les bicarbonates, entre 3,5 et 30 mmol/l pour l'urée, entre 20 et 1000 μmol/l pour la créatinine. Pour la répétabilité, les C.V. (coefficient de variations) se sont situés entre 0,37% et 4,9%, pour la reproductibilité, les C.V. ont varié de 0,88% à 5,92%.

La comparaison des valeurs observées avec des valeurs de consensus obtenues à partir de deux programmes interlaboratoires de contrôle de qualité, a mis en évidence une excellente corrélation pour l'ensemble des six paramètres.

Pendant la période d'évaluation, les différents problèmes rencontrés, relatifs à la fabrication et au mouvement mécanique furent résolus par les constructeurs.

L'étude de l'imprécision et la confrontation des résultats aux valeurs provenant d'un contrôle de qualité indique que le Chemispek J 200 analyse les paramètres sodium, potassoum, chlorures, biocarbonates, urée et créatinine avec un excellent degré de précision et d'exactitude.

### Beurteilung der Leistungen des Chemispek J 200 in einem Spitallabor

G. Dale *et al.*

Der Chemispek J 200 ist ein neulich eingeführter, kontinuierlicher Multikanal-Autoanalysator. Er umfasst mehrere miteinander verbundene Module: Probenahme, Getriebe und Pumpen, Registriergerät, Mikroprozessor, Drucker, Kassettengerät und Netzteil. Ein 6-Kanal Gerät wird für die Analyse von Natrium, Kalium, Chlor, Bikarbonat, Harnstoff und Kreatinin benützt unter Beachtung eines Durchsatzes von 120 Proben pro Stunde mit ungefähr 140 μl Probenvolumen. Die benützte Chemie umfasst Flammenphotometrie für Natrium und Kalium, Thiocyanat-Nitrat für Chlor, Kresolrot für Bikarbonat, Diacetylmonoxim für Harnstoff und alkalisches Pikrat für Kreatinin. Der Mikroprozessor standardisiert, berechnet, korrigiert für Drift und Verschleppung und der 6-Kanal Schreiber überwacht die Kurven. Die Probenkonzentrationen umfassen den Bereich von 100-154 mmol/l für Natrium, 2–10 mmol/l für Kalium, 84–130 mmol/l für Chlorid, 10–40 mmol/l für Bikarbonate, 3,5–30 mmol/l für Harnstoff und 20–1000 μmol/l für Kreatinin. Innerhalb einer Serie betrug die Genauigkeit zwischen 0,37 und 4,9%; von Serie zu Serie lag sie zwischen 0,88 und 5,92%. Ein Vergleich der erhaltenen Resultate mit den Resultaten von 2 externen Qualitäts-Vergleichsmethoden zeigte ausgezeichnete Uebereinstimmung für alle sechs Chemien. Während der Evaluation traten eine Reihe von mechanischen und konstruktiven Problemen auf, die vom Hersteller behoben wurden. Genauigkeit und vergleichbare Qualitätsdaten deuten an, dass der Chemispek J 200 die zu untersuchenden Substanzen (Natrium, Kalium, Chlor, Bikarbonat, Harnstoff, Kreatinin) mit ausgezeichneter Präzision und Genauigkeit bestimmt.

---

### Page 125

### An evaluation of the Corning 902 direct potentiometric sodium/potassium analyser

P. Bijster *et al.*

The authors evaluated the performance of the Corning 902 direct potentiometric sodium/potassium analyser. Intra-assay precisions for whole blood and plasma were found to be about 0·5% or less, both for sodium and potassium. Inter-assay precisions, measured with aliquots of a frozen serum pool over an 18-day period, were 0·3% for sodium and 1·0% for potassium; and inter-assay precision measured with aliquots of a frozen control serum was 0·6% for sodium and 1·7% for potassium. Linearity, both for sodium (concentration range 100–195 mmol/l) and potassium (concentration range 2·0–9·5 mmol/l) was good and the carry-over was less than 0·1%. The Corning 902 gave slightly higher values than flame photometry for both sodium and potassium. pH had little influence on the sodium and potassium concentrations in the pH range normally found in plasma or serum. The mean difference between the sodium and potassium concentrations in whole blood and plasma ($N = 50$) were +0·7 mmol/l and −0·02 mmol/l, respectively. After a standby period the repeated measurement of a sample gave displayed concentrations that tended to drift towards a lower constant value. The difference between the concentration of the sample measured first and the steady-state concentration was about 3% for potassium and was not significant for sodium, because sodium concentration is displayed in three digits.

### Evaluation de l'analyseur de sodium potassium Corning 902 utilisant la potentiometrie directe

P. Bijster *et al.*

Les auteurs int évalué les performances de l'analyseur de sodium/potassium Corning 902 utilisant la potentiométrie directe. La répétabilité pour un sang total et un plasma a été trouvée égale ou inférieur à un C.V. de 0,5% pour le sodium et le potassium. La reproductibilité mesurée sur une période de 18 jours à l'aide d'aliquots provenant d'un pool de sérum congelé a donné un C.V. de 0,3% pour le sodium, et de 1% pour le potassium; dans les mêmes conditions, avec un sérum de contrôle congelé, les résultats one été C.V = 0,6% pour le sodium, et C.V = 1,7% pour le potassium. La linéarité pour le sodium (100 à 195 mmol/l) et le potassium (2 à 9,5 mmol/l), a été trouvée bonne, et la contamination a été trouvée inférieure à 0,1%. Le Corning 902 a donné des valeurs légèrement supérieures à celles de la photométrie de flamme, à la fois pour le sodium, et le potassium. Il a été démontré que le pH influençait légèrement les concentrations de concentrations de sodium et de potassium pour une plage de pH normalement rencontrée dans le plasma ou le sérum. La moyenne des différences entre le sang total et le plasma a été trouvée pour un effectif .de 50, de +0,7 mmol/l pour le sodium, et

−0,02 mmol/l pour le potassium. Espacée d'une période d'attente, la répétition de la mesure d'un même échantillin a montré une légère dérive vers le bas, cette différence entre ces deux mesures est égale à 3% pour le potassium, et est non significative pour le sodium, l'affichage à trios chiffres expliquant la non-visualisation de la légère dérive pour ce dernier.

**Evaluation des Corning 902 Direktpotentiometrie-Na/K-Analysators**

P. Bijster *et al.*

Die Autoren evaluierten die Leistungsfähigkeit des Corning 902 Direktpotentiometrie-Na/K-Analysators. Die innerhalb einer Messreihe gefundenen Genauigkeiten für Gesamtblut und Plasma waren ungefähr 0,5% oder weniger, sowohl für Natrium als auch für Kalium. Die Genauigkeiten über verschiedene Messreihen, gemessen an einem Aliquot von einem gefrorenen Serumvorrat über eine Periode von 18 Tagen, betrugen 0,3% für Natrium und 1,0% für Kalium. Die Genauigkeit gemessen an den Aliquoten eines gefrorenen Kontrollserums betrug 0,6% für Natrium und 1,7% für Kalium. Die Linearität für Natrium (Konzentrationsbereich 100–195 mmol/l) und Kalium (Konzentrationsbereich 2,0–9,5 mmol/l) war gut und die Verschleppung betrug weniger als 0,1%. Der Corning 902 ergab leicht höhere Werte als mit Flammenphotometrie, sowohl für Natrium als auch Kalium. Der pH hatte wenig Einfluss auf die Natrium- und Kaliumkonzentrationen im pH-Bereich, der normalerweise in Plasma oder Serum anzutreffen ist. Die mittleren Unterschiede zwischen den Natrium- und den Kaliumkonzentrationen in Gesamtblut und Plasma ($N = 50$) betrugen +0,7 mmol/l und −0,02 mmol/l. Nach einer Anwärmperiode ergaben wiederholte Messungen einer Probe angezeigte Konzentrationswerte, die langsam zu einem tieferen, konstanten Wert drifteten. Die Differenz zwischen dem ersten gemessenen Wert und dem Endwert betrug ca. 3% für Kalium, war aber insignifikant für Natrium, weil die Natriumonzentration nur 3-stellig angezeigt wird.

### Page 129

**A report on the performance of the Gilford EIA 50 automated micro-ELISA processor**

R. Goodburn *et al.*

The performance of the Gilford (Corning Ltd) EIA 50 automated ELISA (enzyme-linked immunosorbent assay) processor has been investigated. The time-consuming and technically demanding tasks involved in the micro-ELISA technique are performed well by this processor. It uses specially produced micro-ELISA cuvettes rather than the usual microtitre plates; difficulties were experienced with these cuvettes initially, but improved manufacturing methods have overcome these difficulties. The improved cuvettes have enabled the system to work within the limits of accuracy and precision required for quantitative micro-ELISA methods.

**Etude des performances du Gilford EIA 50 Automatisé avec le Micro-ELISA processor**

R. Goodburn *et al.*

Les performances du Gilford EIA 50 automatisé par le 'Micro-ELISA (enzyme-linked immunosorbent assay) processor' ont été testées. Les contraintes de temps et les différentes étapes de la technique Micro-ELISA sont pilotées par le microprocesseur. Cet appareil utilise des cuvettes Micro-ELISA spéciales, à la place des plaques de microtitration habituelles. Les difficultés qui étaient apparues initialement avec ces cuvettes, ont disparu après une modification de leur mode de fabrication. Les cuvettes modifiées permettent le fonctionnement du système dans des limites d'exactitude et de précision admises pour des techniques quantitatives Micro-ELISA.

**Bericht über Ergebnisse mit dem Gilford EIA 50 automatisierten Mikro-ELISA Prozessor**

R. Goodburn *et al.*

Die Leistungsfähigkeit des Gilford (Corning Ltd) EIA 50 automatisierten ELISA (enzyme-linked immunosorbent assay) Prozessors wurde untersucht. Die zeitraubenden und technisch anspruchsvollen Aufgaben, die in die Mikro-ELISA-Technik involviert sind, werden durch diesen Prozessor gut ausgeführt. Das Gerät benützt anstelle der üblichen Mikrotiter-Platten speziell hergestellte Mikro-ELISA-Cuvetten; die mit diesen Cuvetten anfänglich aufgetretenen Schwierigkeiten wurden mit verbesserten Herstellmethoden überwunden. Die verbesserten Cuvetten ermöglichten dem System innerhalb der Genauigkeits- und Präzisionsgrenzen zu arbeiten, die für die quantitative Mikro-ELISA-Methoden erforderlich sind.

### Page 135

**Thermostatic regulation of blood samples in blood gas analysers: results and improved method applied to 11 different models**

B. Dingeon *et al.*

A simple technique for precisely measuring the temperature of the blood sample in blood gas analysers is described. The method needs no modification of the instrument and can be immediately tried on all manual or automatic analysers. Eleven instruments were tested and the results of lag time and accuracy are discussed and presented.

**Thermostatisation d'èchantillons de sang pour analyseur de gaz dans le sang: résultats avec méthode améliorée, appliquée sur 11 modéles différents**

B. Dingeon *et al.*

Une technique simple pour la mesure exacte de la temperaturedans des èchantillons de sang pour analyseur de gaz dans le sang est décrite. la méthode ne demande aucune modification de l'instrument et elle peut immédiatement être testée sur tous les analyseurs manuels et automatiques. La methode a été vérifiée sur onze instruments et les résultats pour les temps de rétention et les précisions sont donnés et discutés.

**Thermostatisierung von Blutproben in Blutgasanalysatoren: Resultate mit verbesserter Methode, angewandt auf 11 verschiedene Modelle**

B. Dingeon *et al.*

Eine einfache Technik für die genaue Temperaturmessung von Blutpoben in Blutgasanalysatoren ist beschrieben. Die Methode erfordert keine Modifikation des Instruments und kann unmittelbar auf allen manuellen und automatischen Analysatoren ausprobiert werden. Auf elf Instrumenten wurde die methode geprüft und die Resultate für Verzögerungszeit und Genauigkeit werden angeführt und diskutiert.

### Page 139

### Present documents on standardization of connection parts (interfaces and ports)

U. Schmidt

With the increasing use of computer data processing and control in analytical laboratories, particularly in clinical laboratories, the need for defining interface conditions is becoming increasingly important. This paper discusses the requirements for a standard interface and outlines the current status of the documentation available for defining such an interface. The need for work in additional areas to complete the documentation is also considered.

### Documents actuels sur la standardisation des éléments de connexion (interfaces et connecteurs)

U. Schmidt

Avec l'emploi grandissant des ordinateurs pour la gestion des données et des contrôles dans les laboratoires d'analyses, et plus particulièrement dans les laboratoires d'analyses médicales. Il devient de plus en plus important de définir des conditions d'interfaçage.

Cet article passe en revue les conditions requises pour fabriquer une interface standard et détermine les informations indispensables pour définir une telle interface.

### Heutige Unterlagen zur Standardisierung von Anschlüssen (Schnittstellen und Datenein- und Datenausgänge)

U. Schmidt

Wegen der zunehmenden Benützung von Rechnern für die Datenverarbeitung und Steuerung in analytischen Laboratorien, speziell klinischen Laboratorien, steigt auch die Notwendigkeit für eine Schittstellendefinition. Die vorliegende Arbeit behandelt die Erfordernisse für eine Standardschnittstelle und beschreibt den gegenwärtigen Stand der für die Definition solcher Schnittstellen verfügbaren Dokumentation.

Ebenso wird die Notwendigkeit für eine Bearbeitung zusätzlicher Aspekte zur Vervollständigung der Dokumentation behandelt.

NEW FROM SHIMADZU

GC–8A Series GAS CHROMATOGRAPHS

*Dual FID With quartz jets and cylindrical collectors

*Dual TCD Constant current type with differential (semi-diffusion) flow and preamplifier. Full filament protection.

*Column Oven Programmable or Isothermal. Low thermal mass for quick analytical cycling. Glass, metal or capillary columns. Two stage overheat protection.

*Independent heating of Injection Ports/Detectors.

*Digital temperature setting.

*On column injection.

*Full flow controls (mass flow controllers are standard on carrier gases).

*Plus SHIMADZU's world renowned reputation for exceptional reliability and high performance.

*At a price you can afford.

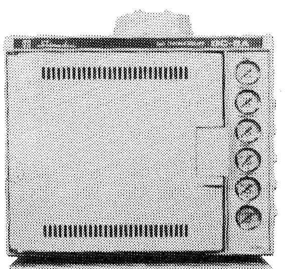

We are always available to advise on the most suitable equipment for any application and to demonstrate in a user's laboratory. We particularly welcome visitors to our headquarters where we can demonstrate the practical application of our comprehensive range of scientific instrumentation.

Sunderland House, Station Road, Hetton, Houghton-le-Spring, Tyne & Wear DH5 0AT England Telephone: Hetton (0783) 260452 & 260433 Telex: 53689

DYSON INSTRUMENTS

Circle No. 3 on Reader Enquiry Card

### Reader Enquiry Service

A new feature in *Journal of Automatic Chemistry*, introduced with this issue, is a Reader Enquiry Service.

Each advertisement and each editorial item in the Product News section carries a number, which corresponds with a number on the Reader Enquiry Card at the back of this issue.

If there are any items on which you would like further information, circle the relevant number on the card, indicate the volume and issue number, fill in your name and full address, and return the card to Taylor & Francis Ltd. Your card will then be forwarded to the company/ies concerned for action.

*Through using the Reader Enquiry Service, you can obtain information on a variety of products through one simple operation.*

